# Standardized cannabis in multiple sclerosis: a case report

**DOI:** 10.1186/1757-1626-3-7

**Published:** 2010-01-06

**Authors:** Paul Hornby, Manju Sharma

**Affiliations:** 1 Department of Medical Cannabis Research, The Green Cross Society of BC, 2127, Kingsway, Vancouver, B.C., V5N 2T4, Canada; 2 Department of Pathology and Laboratory Medicine, Heather Pavilion, Vancouver, General Hospital, Vancouver, B.C., Canada

## Abstract

A 52 year old female suffering from severe progressive multiple sclerosis was administered quantifiable amounts of standardized cannabis and monitored over the period of one year, while providing daily pain charts and records of her condition. An average daily intake of 500 mg of Tetrahydrocannabinol as cannabis was required to achieve a desired quality of life.

## Introduction

Multiple Sclerosis (MS) is a difficult disease both to diagnose and to treat. Diagnosis often requires multiple visits to the physician and it may take years before MS is diagnosed, typed and treated.

There is no cure for MS. Treatment is based on powerful immune system suppressants, mainly steroids and various types of Interferon, although others may be used as well. In addition, many types of medications including anticholinergics, antispasmodics, benzodiazepines and opiates are used to manage the muscle spasms, bladder incontinence issues and nerve pain that may be associated with MS. They do however prevent recurrence and slow progression of the illness. Prevention of MS through vitamin D supplementation is an intriguing possibility.

In the study described here we trace the cannabis use of an MS patient over the course of one year. High Pressure Liquid Chromatography (HPLC) was performed to quantify cannabis. The subject made significant improvement with better pain control, decreased muscle spasms and general quality of life. The case described here is one of many observed at the Green Cross Society of B.C.

## Case presentation

The subject in this study, a 52-year-old female, is a member of the Green Cross Society which is a non-profit, organization, dedicated to supplying quality controlled, standardized cannabis to its qualified members. The participant involved in this year-long study was chosen primarily because she had an attentive, full-time, caregiver, who had tracked her illness since its beginning in the late 1970's. It was the caregiver that first noticed that cannabis was beneficial in relieving her symptoms. This however, was sometime after the disease was diagnosed.

Initially she was subjected to a 10-day regimen of ACTH, plus various muscle relaxants and anxiolytics. Intermittent ACTH treatment occurred until 1985 when Magnetic Resonance Imaging confirmed her diagnosis with MS. She then began daily injections of Copaxone, which gave benefits including reduction of muscle spasms and a degree of pain management. In addition, she would also smoke cannabis to further alleviate symptoms.

Initial symptoms had included numbness of the right side of the lip, and a depressed gag reflex that made swallowing difficult. These symptoms had been present for a decade prior to the suspicion and diagnosis of MS. In 1983, she began experiencing extreme pain in her lower lumbar region that radiated to her left foot, affecting her ability to walk. When she first came to the Society in 2007, she complained of chronic pain, tremor, difficulty in walking and a severe constant pain in her left foot.

Through the Green Cross Society, the subject received advice on the best cannabis strain selection for her symptoms plus options on means of administration and dosage regimens. For the following year she ingested cannabis that had been tested for concentrations of the most abundant cannabinoids including Delta-9 Tetrahydrocannabinol (THC), Cannabidiol (CBD) and Cannabinol (CBN). Her caregiver provided daily (email) pain charts, plus ingested medications, and a description of the subject's general well being throughout the study.

## Discussion

**Figure 1:**
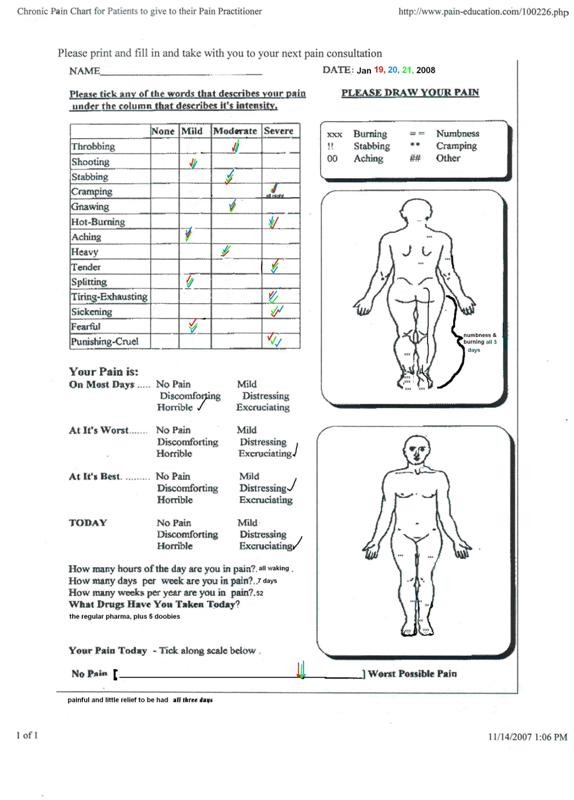
**Example pain chart from early in the study**.

**Figure 2:**
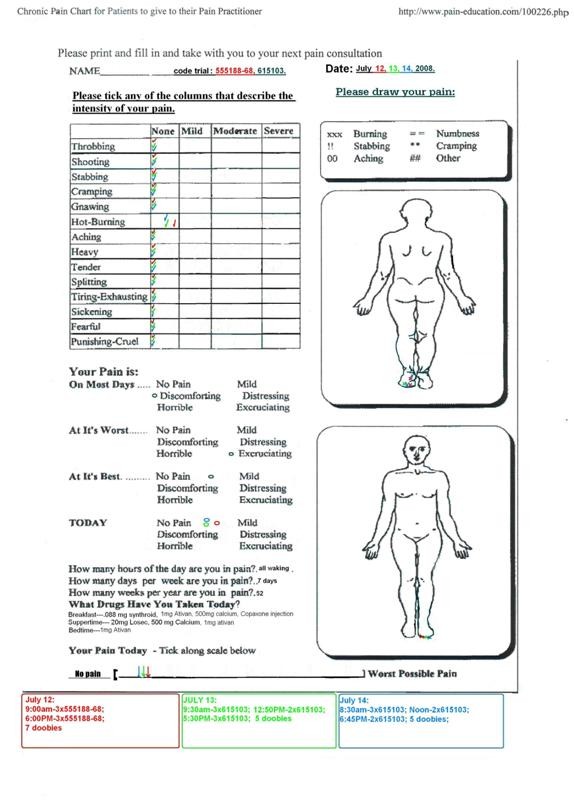
**Example pain chart from study mid-point**.

Results were assessed through daily dialogue with the subject's caregiver, review of accumulated pain charts, plus the subject's testimony. An almost immediate improvement was seen in the patient's condition when she began administering the standardized oral preparations. Pain scores were reduced from an 8 - 10 to 1 - 2 over the period of the first month during which time the optimal dosing regimen was established (Figures 1 and 2: example pain charts). Greatest benefit for pain and tremor was achieved consuming 6-8, 50 mg capsules per day, dosing at 4-hour intervals. The subject also consumed an average of 4-6 cannabis cigarettes per day, for breakthrough pain and/or mood enhancement. Measurement of the mean THC concentration of these cigarettes was 172 ± 26 n = 30, that would equate to a dosage of 25 mg of THC per cigarette (0.5 grams per cigarette with roughly 70% loss to atmosphere) [1], adding another 125 mg to her daily consumption, totaling between 400 and 500 mg per day of THC. The average relative amounts of CBD and CBN are roughly 3% of that found for THC. A significant improvement in pain levels, tremor and general well being were observed. Functionally, the patient went from a state of virtual incapacitation to one where she can dress, go for walks and do some gardening: a dramatic improvement over the course of one year.

The presence of CBD and CBN, although claiming no psychoactive effect, appear to modulate the binding of THC to its receptor and thus alter the efficacy of the preparation. The relative ratio of these three cannabinoids is determined by the specific strain of cannabis. Over the course of the study year it was found that the subject experienced optimal relief with strains containing relatively high CBD to THC ratio of 4-6%, a high THC concentration and relatively low CBN amount. Observations made with the study subject and, indeed, verified day-to-day at the Society is that those managing chronic pain prefer these ratios.

The seemingly high amounts of THC (approximately 500 mg/day) required by the subject to manage her symptoms are often observed with persons of her genealogy (Scottish). An earlier publication on the effects of standardized cannabis and chronic pain management describes a similar tolerant response [2]. A phenomenon often witnessed at the Society is persons with Celtic genealogy require 3 -5 five times greater dosage than those of Middle European or other descent.

The only observed unwanted side effects were seen when the strain used in the oral capsules was changed to one that did not provide the same pain or anti-tremor relief as the strain provided at an earlier time. Simply put, there was some discomfort experienced in changing strains. Since the subject was receiving the medication by mail, from time to time, she would run out of the oral preparation at which time her symptoms would worsen to their pre-regimen state within days. Recent tests of liver functions proved normal.

## Conclusions

The established non-toxicity of cannabis and non-addictive properties [3] make it an excellent candidate for treating the symptoms of numerous illnesses. The case described here is one of many observed at the Green Cross Society of B.C.

## Footnote HPLC

High pressure liquid chromatography, using a Hewlett-Packard (Agilent) 1090 Series II binary pumping system with a 79883a diode array detector, with primary absorbance at 219 nm, was conducted. Mobile phase at 1 ml/min was isocratic with 14% aqueous (1:25:974 phosphoric acid: acetonitrile: distilled water) and 86% organic phase (acetonitrile). The column was a Zorbax C18 reverse phase 4.6 mm × 25 cm. Samples were prepared by the method modified from Health Canada for the preparation of hemp samples for HPLC analysis [4]. Calibration curves were run for a number of commercial standards (Sigma) and averages made. 0.1 gram of dry (dryness determination appendix 1) cannabis was suspended in 10 ml THF and sonicated for 3 minutes then passed through a 0.45 micron nylon syringe filter into a 1 ml HPLC sample vial. Under these conditions suitable chromatography is achieved for the three most abundant cannabinoids present in the samples provided to the Society's members.

## Abbreviations

CBD-A: Cannabidiolic acid; CBD: Cannabidiol; CBN-A: Cannabinolic Acid; CBN: Cannabinol; THC-A: Tetrahydrocannabinolic Acid; THC: Delta-9 tetrahydrocannabinol; THF: tetrahydrofuran; HPLC: high performance liquid chromatography.

## Consent

Written informed consent was obtained from the patient for publication of this case report. A copy of the written consent is available for review by the Editor-in-Chief of this journal.

## Competing interests

The authors declare that they have no competing interests.

## Authors' contributions

PH conducted data collection and analysis, plus manuscript draft preparation. MS, interpreted data and edited the manuscript.
